# Supplementary material for: Iterative improvement in the automatic modular design of robot swarms
Source: PeerJ Comput Sci. 2020 Dec 7;6:e322. doi: 10.7717/peerj-cs.322 (PMC7924708; doi:10.7717/peerj-cs.322)
Supplement: Supplemental Information 3 [file peerj-cs-06-322-s003.zip › argos3/doc/api/standalone/a00661_source.html]

ARGoS: plugins/simulator/visualizations/qt-opengl/qtopengl\_widget.h Source File


- Main Page
- Related Pages
- Namespaces
- Classes
- Files

- File List
- File Members

# plugins/simulator/visualizations/qt-opengl/qtopengl\_widget.h

Go to the documentation of this file.

```
00001 
00007 #ifndef QTOPENGL_WIDGET_H
00008 #define QTOPENGL_WIDGET_H
00009 
00010 namespace argos {
00011    class CQTOpenGLWidget;
00012    class CQTOpenGLMainWindow;
00013    class CSpace;
00014    class CSimulator;
00015    class CQTOpenGLBox;
00016    class CQTOpenGLUserFunctions;
00017    class CPositionalEntity;
00018    class CControllableEntity;
00019    class CEmbodiedEntity;
00020 }
00021 
00022 #include <argos3/plugins/simulator/visualizations/qt-opengl/qtopengl_camera.h>
00023 #include <argos3/core/simulator/entity/entity.h>
00024 #include <argos3/core/utility/datatypes/datatypes.h>
00025 #include <QOpenGLWidget>
00026 #include <QOpenGLFunctions>
00027 #include <QOpenGLTexture>
00028 
00029 #ifdef __APPLE__
00030 #include <glu.h>
00031 #else
00032 #include <GL/glu.h>
00033 #endif
00034 
00035 namespace argos {
00036 
00037    /****************************************/
00038    /****************************************/
00039 
00040    class CQTOpenGLOperationDrawNormal : public CEntityOperation<CQTOpenGLOperationDrawNormal, CQTOpenGLWidget, void> {
00041    public:
00042       virtual ~CQTOpenGLOperationDrawNormal() {}
00043    };
00044 
00045    class CQTOpenGLOperationDrawSelected : public CEntityOperation<CQTOpenGLOperationDrawSelected, CQTOpenGLWidget, void> {
00046    public:
00047       virtual ~CQTOpenGLOperationDrawSelected() {}
00048    };
00049 
00050 #define REGISTER_QTOPENGL_ENTITY_OPERATION(ACTION, OPERATION, ENTITY)   \
00051    REGISTER_ENTITY_OPERATION(ACTION, CQTOpenGLWidget, OPERATION, void, ENTITY);
00052 
00053    /****************************************/
00054    /****************************************/
00055 
00056    class CQTOpenGLWidget : public QOpenGLWidget, protected QOpenGLFunctions {
00057 
00058       Q_OBJECT
00059 
00060     public:
00061 
00065       struct SFrameGrabData {
00066          bool Grabbing;     // true when grabbing
00067          QString Directory; // output directory
00068          QString BaseName;  // frame file basename
00069          QString Format;    // output file format
00070          SInt32 Quality;    // output quality [0-100]
00071 
00072          SFrameGrabData() :
00073             Grabbing(false),
00074             Directory("."),
00075             BaseName("frame_"),
00076             Format("png"),
00077             Quality(-1) {}
00078 
00079          void Init(TConfigurationNode& t_tree);
00080       };
00081 
00085       struct SSelectionInfo {
00086          bool IsSelected;         // true when a robot is selected
00087          size_t Index;            // the index of the selected robot
00088 
00089          SSelectionInfo() :
00090             IsSelected(false),
00091             Index(0) {}
00092       };
00093 
00094    public:
00095 
00102       CQTOpenGLWidget(QWidget* pc_parent,
00103                       CQTOpenGLMainWindow& c_main_window,
00104                       CQTOpenGLUserFunctions& c_user_functions);
00108       virtual ~CQTOpenGLWidget();
00109 
00115       virtual void initializeGL();
00116 
00120       virtual void paintGL();
00121 
00126       CRay3 RayFromWindowCoord(int n_x,
00127                                int n_y);
00128 
00132       CVector3 GetWindowCoordInWorld(int n_x,
00133                                      int n_y);
00134 
00139       CEntity* GetSelectedEntity();
00140 
00145       void SelectEntity(CEntity& c_entity);
00146 
00151       void DeselectEntity();
00152 
00158       void SelectInScene(UInt32 un_x,
00159                          UInt32 un_y);
00160 
00164       void DrawEntity(CPositionalEntity& c_entity);
00165 
00169       void DrawEntity(CEmbodiedEntity& c_entity);
00170 
00174       void DrawRays(CControllableEntity& c_entity);
00175 
00179       void DrawBoundingBox(CEmbodiedEntity& c_entity);
00180 
00184       inline virtual int heightForWidth(int w) const {
00185          return (w * 3) / 4;
00186       }
00187 
00191       inline virtual QSize sizeHint() const {
00192          return QSize(1024,768);
00193       }
00194 
00198       inline virtual QSize minimumSize() const {
00199          return QSize(320,240);
00200       }
00201 
00205       inline CQTOpenGLUserFunctions& GetUserFunctions() {
00206          return m_cUserFunctions;
00207       }
00208 
00212       inline CQTOpenGLCamera& GetCamera() {
00213          return m_cCamera;
00214       }
00215 
00219       inline SFrameGrabData& GetFrameGrabData() {
00220          return m_sFrameGrabData;
00221       }
00222 
00226       inline void SetInvertMouse(bool b_InvertMouse) {
00227           m_bInvertMouse = b_InvertMouse;
00228       }
00229 
00230    signals:
00231 
00236       void StepDone(int n_step);
00237 
00243       void ExperimentDone();
00244 
00249       void EntitySelected(size_t un_index);
00250 
00255       void EntityDeselected(size_t un_index);
00256 
00257    public slots:
00258 
00264       void PlayExperiment();
00265 
00270       void FastForwardExperiment();
00271 
00277       void PauseExperiment();
00278 
00282       void StepExperiment();
00283 
00288       void ResetExperiment();
00289 
00293       void SetDrawFrameEvery(int n_every);
00294 
00298       void SetGrabFrame(bool b_grab_on);
00299 
00304       void SetCamera(int n_camera);
00305 
00310       void SetCameraFocalLength(double f_length);
00311 
00315       void KeyPressed(QKeyEvent* pc_event);
00316 
00320       void KeyReleased(QKeyEvent* pc_event);
00321 
00322    protected:
00323 
00324       void DrawScene();
00325       void DrawArena();
00326       void DrawAxes();
00327 
00328       virtual void timerEvent(QTimerEvent* pc_event);
00329       virtual void mousePressEvent(QMouseEvent* pc_event);
00330       virtual void mouseReleaseEvent(QMouseEvent* pc_event);
00331       virtual void mouseMoveEvent(QMouseEvent* pc_event);
00332       virtual void keyPressEvent(QKeyEvent* pc_event);
00333       virtual void keyReleaseEvent(QKeyEvent* pc_event);
00334       virtual void resizeEvent(QResizeEvent* pc_event);
00335       void reactToKeyEvent();
00336 
00337    private:
00338 
00340       CQTOpenGLMainWindow& m_cMainWindow;
00342       CQTOpenGLUserFunctions& m_cUserFunctions;
00343 
00345       SInt32 nTimerId;
00347       bool m_bFastForwarding;
00349       SInt32 m_nDrawFrameEvery;
00351       SInt32 m_nFrameCounter;
00352 
00354       bool m_bMouseGrabbed;
00356       bool m_bShiftPressed;
00358       QPoint m_cMouseGrabPos;
00360       bool m_bInvertMouse;
00362       SSelectionInfo m_sSelectionInfo;
00363 
00365       CSimulator& m_cSimulator;
00367       CSpace& m_cSpace;
00368 
00370       bool m_bUsingFloorTexture;
00372       QOpenGLTexture* m_pcFloorTexture;
00374       QOpenGLTexture* m_pcGroundTexture;
00375 
00377       GLfloat* m_pfLightAmbient;
00379       GLfloat* m_pfLightDiffuse;
00381       GLfloat* m_pfLight0Position;
00383       GLfloat* m_pfLight1Position;
00384 
00386       GLuint m_unArenaList;
00388       GLuint m_unFloorList;
00390       GLuint* m_punSelectionBuffer;
00391 
00393       CQTOpenGLCamera m_cCamera;
00395       SFrameGrabData m_sFrameGrabData;
00396 
00398       enum EDirection {
00399          DIRECTION_UP = 1,
00400          DIRECTION_DOWN,
00401          DIRECTION_LEFT,
00402          DIRECTION_RIGHT,
00403          DIRECTION_FORWARDS,
00404          DIRECTION_BACKWARDS,
00405       };
00406 
00408       QMap<EDirection, int> m_mapPressedKeys;
00409 
00410       CRay3 m_cSelectionRay;
00411    };
00412 
00413 }
00414 
00415 #endif
```

---

Generated on 10 Jul 2018 for ARGoS by 
 1.6.1
